# Intermedin Inhibits DNA Damage-Promoted Senescent Phenotype Transition of Vascular Smooth Muscle Cells in Aorta by Activating NAMPT/PARP1 in Mice

**DOI:** 10.3390/ph18101503

**Published:** 2025-10-07

**Authors:** Deng-Ren Ji, Yao Chen, Han-Xu Zhu, Shi-Meng Liu, Ning Wu, Ya-Rong Zhang, Jie Zhao, Yan-Rong Yu, Mo-Zhi Jia, Ling Han, Chao-Shu Tang, Lei-Lei Chen, Ye-Bo Zhou, Yong-Fen Qi

**Affiliations:** 1 Laboratory of Cardiovascular Bioactive Molecule, School of Basic Medical Sciences, Peking University, Beijing 100083, China; 1510305113@pku.edu.cn (D.-R.J.); fexis@hsc.pku.edu.cn (S.-M.L.); yarong@bjmu.edu.cn (Y.-R.Z.); zhaojie828@bjmu.edu.cn (J.Z.); 2 Key Laboratory of Molecular Cardiovascular Science, Ministry of Education, Peking University Health Science Center, Beijing 100083, China; tangchaoshu@263.net.cn; 3 Department of Pathogen Biology, School of Basic Medical Sciences, Peking University, Beijing 100083, China; yuyr@bjmu.edu.cn (Y.-R.Y.); mzhjia@bjmu.edu.cn (M.-Z.J.); 4 Department of Physiology, School of Basic Medical Sciences, Chongqing Medical University, Chongqing 400016, China; chenyao_basics@cqmu.edu.cn; 5 Department of Physiology, Nanjing Medical University, Nanjing 211166, China; zhuhx200110@163.com; 6 Department of Obstetrics and Gynecology, Peking University First Hospital, Beijing 100034, China; wuning100@163.com; 7 Department of Cardiology, Fu Xing Hospital, Capital Medical University, Beijing 100038, China; hling966@sina.com; 8 Department of Cardiology, The First Affiliated Hospital of Nanjing Medical University, Nanjing 210029, China; chenlei19762002@163.com

**Keywords:** intermedin, senescent phenotype transition, vascular smooth muscle cells, DNA damage, PARP1, NAD

## Abstract

**Background and aims**: The senescent phenotype transition of vascular smooth muscle cells (VSMCs) is a crucial risk factor for the occurrence and development of vascular diseases. Intermedin (IMD) has various protective effects on cardiovascular diseases. In this study, we aimed to explore the role and the related mechanism of IMD in the senescent phenotype transition of VSMCs of aorta in mice. **Methods**: The senescent phenotype transition of VSMCs was induced by angiotensin II (Ang II) administered by mini-osmotic pumps in *Adm2*^fl/fl^ and *Adm2*^fl/fl^*Tag*Cre mice. Mouse VSMCs from aorta were used in in vitro experiments. **Results**: The aortic mRNA level of *IMD*, namely *Adm2,* was significantly decreased in Ang II-treated mice. Senescence-associated β-galactosidase activity and protein expressions of p16 and p21 were increased in the aortas of *Adm2*^fl/fl^*Tag*Cre mice, which were further elevated in Ang II-treated *Adm2*^fl/fl^*Tag*Cre mice. In addition, *Adm2* deficiency in VSMCs further increased the protein expressions of DNA damage markers including 53BP1 and γH2AX in aortas of *Adm2*^fl/fl^*Tag*Cre mice, and Ang II treatment increased their levels in aortas of *Adm2*^fl/fl^*Tag*Cre mice or in VSMCs. However, Ang II-induced increases in senescence-associated proteins and DNA damage markers could be mitigated by the administration of IMD in vitro. Mechanistically, IMD increased intracellular NAD^+^ by activating nicotinamide phosphoribosyl transferase (NAMPT), followed by enhancing poly (ADP-ribose) polymerase-1 (PARP1) activity. Inhibitors of PARP1 or NAMPT effectively blocked the beneficial role of IMD in the DNA damage of VSMCs. **Conclusions**: IMD alleviates DNA damage partially by activating NAMPT/PARP1, thereby inhibiting the senescent phenotype transition of VSMCs of aorta, which might shed new light on the prevention of vascular aging.

## 1. Introduction

The aortic wall is composed of three structurally distinct layers: intima, media, and adventitia. The arterial media is usually the thickest layer; it provides support for the vessel and alters vessel diameter to regulate blood flow and blood pressure. Vascular smooth muscle cells (VSMCs) are the main cells of tunica media and play an important role in maintaining vascular function depending on its systolic phenotype [1]. Moreover, the abnormal VSMC’s plasticity and signaling are highly relevant to early vascular aging [2]. And increasing experimental data indicated that the senescent phenotype transition of VSMCs (from systolic phenotype to senescent phenotype) plays a pivotal role in inducing aging-related vascular remodeling [3,4]. In addition, a previous study found that attenuating angiotensin II (Ang II)-induced VSMCs senescence could effectively alleviate aging-associated vascular remodeling [5]. The VSMCs is an initial and fundamental aging process in which cells permanently withdraw from the cell cycle under the stimulation of a range of endogenous and exogenous stressors such as telomere dysfunction, DNA damage, reactive oxygen species (ROS), and paracrine signals and undergo distinctive phenotypic changes [6]. It is necessary to inhibit the senescent phenotype transition of VSMCs in the prevention and treatment of vascular aging and related diseases.

DNA damage and genome instability play a crucial role in the initiation and progression of cellular senescence [7]. DNA damage caused by endogenous and exogenous stimuli triggers the DNA damage response (DDR), which in turn affects cell fate, repair, or senescence [8]. To deal with DNA damage, a mature DNA repair system has evolved, which can restore the correct base sequence in most cases. However, disturbance of the DNA repair system may lead to sustained DNA damage [7]. In response to sustained DNA damage, cells undergo phenotypic transitions such as the generation of a bioactive secretome, namely the senescence-associated secretory phenotype, represented by the increased protein expressions, such as pro-inflammatory cytokines, chemokines, and growth factors [9]. The transcriptional activation of p16 and p21, two cyclin-dependent kinase inhibitors, is also induced by DDR and leads to cell cycle arrest [10]. Therefore, the accumulation of DNA damage over time is a fundamental driver of aging. Attenuation of DNA damage might delay the senescent phenotype transition of VSMCs and then reverse vascular aging.

Intermedin (IMD), also known as adrenomedullin 2 (ADM2), is a secreted peptide belonging to the calcitonin gene-related peptide (CGRP) superfamily [11,12]. The human IMD gene encodes a prepropeptide composed of 148 amino acids, which has a signal peptide at its N-terminus. By proteolytic cleavage at Arg93-Arg94, IMD_1-53_ can be produced from pre-pro-IMD [13]. IMD exerts its biological effects by combining with calcitonin receptor-like receptor (CRLR) and receptor activity modifying protein (RAMP)1/2/3 [11]. It plays an important role in maintaining cardiovascular homeostasis and exerting a potential beneficial role in cardiovascular diseases (CVDs). Increasing evidence shows that IMD has a protective effect on vascular abnormalities, including vascular calcification [14,15] and abdominal aortic aneurysm [16]. Moreover, our previous study found that IMD levels were significantly decreased in aortas of aging rat and senescent VSMCs, and supplementation with exogenous IMD significantly reduced the protein levels of p16 and p21 in aging rat aortas [17], suggesting that IMD might be involved in the regulation of senescent phenotype transition of VSMCs. In this study, we aimed to investigate the role of IMD in Ang II-induced senescent phenotype transition of VSMCs and the possible mechanism involving DNA damage.

## 2. Results

### 2.1. IMD Expression and Ang II-Induced Senescent Phenotype Transition of VSMCs in Aorta of Mice

**Figure 1:**
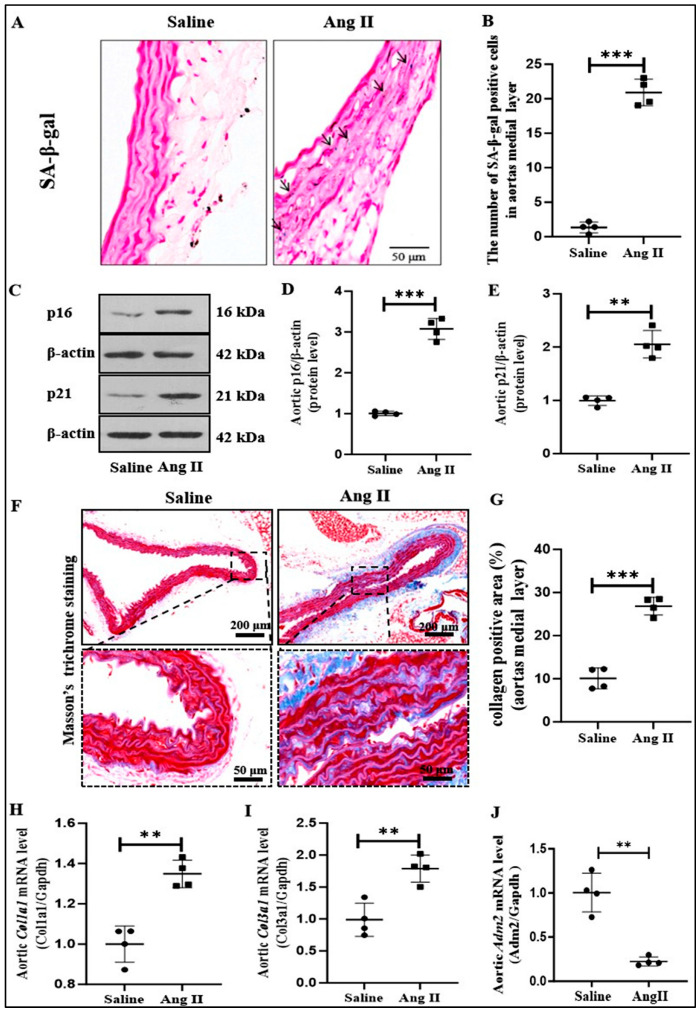
Ang II successfully induced senescent phenotype transition in tunic media of aorta in mice. SA-β-gal staining for β-galactosidase activity (blue) (scale bar = 50 μm) in the mouse thoracic aortas, and the number of positive cells of β-galactosidase-staining under the same vascular cross-sectional area ((**A**,**B**), *n* = 4), the arrow indicates positive staining. Western blot analysis of the protein levels of p16 and p21 and quantification ((**C**–**E**), *n* = 4). Masson’s trichrome staining for collagen (blue) (scale bar = 200 μm, 50 μm) in the mouse thoracic aortas, and quantification of collagen-positive staining in the medial layer ((**F**,**G**), *n* = 4). Quantification of Real-time PCR analysis of mRNA levels of *Col1a1*, *Col3a1*, and *Adrenomedullin 2 (Adm2)* in mouse aortas ((**H**–**J**), *n* = 4). Data are mean ± SD. ** *p* < 0.01, *** *p* < 0.001, the Mann–Whitney test.

In this study, mice with initial changes in senescent phenotype transition of VSMCs in aorta of mice were constructed by Ang II subcutaneous buried pump [17,18]. First, the changes in senescent markers in senescence-associated β-galactosidase (SA-β-gal) activity and protein levels of cyclin-dependent kinase inhibitors p16 and p21 in the aorta of mice were detected. Compared to controls, Ang II-induced membrane SA-β-gal activity was significantly higher in mouse aorta (Figure 1A,B), and protein levels of p16 and p21 were also significantly increased (Figure 1C–E). Vascular fibrosis is one of the features of vascular aging [19], and the area of Ang II-induced aortic fibrosis was significantly increased in mice compared with controls (Figure 1F,G). Real-time PCR results also showed that the aortic mRNA levels of *Col1a1* and *Col3a1* were significantly higher in Ang II-treated mice than in the control group (Figure 1H,I). In conclusion, Ang II-induced senescent phenotype transition of VSMCs in aorta as shown by the increased activity of aging-related markers SA-β-gal, protein levels of p16 and p21 proteins, and fibrosis in aorta of mice, indicated that the senescent phenotype transition of VSMCs in aorta was successfully constructed. Moreover, the mRNA level of *IMD*, namely *Adm2*, in the aortic tunic media of the Ang II-treated group was significantly decreased when compared with the control group (Figure 1J), suggesting that IMD may be involved in the pathogenesis of aortic aging induced by Ang II.

### 2.2. Deficiency in Endogenous IMD Exacerbated Ang II-Induced Senescent Phenotype Transition of VSMCs in Aorta

**Figure 2:**
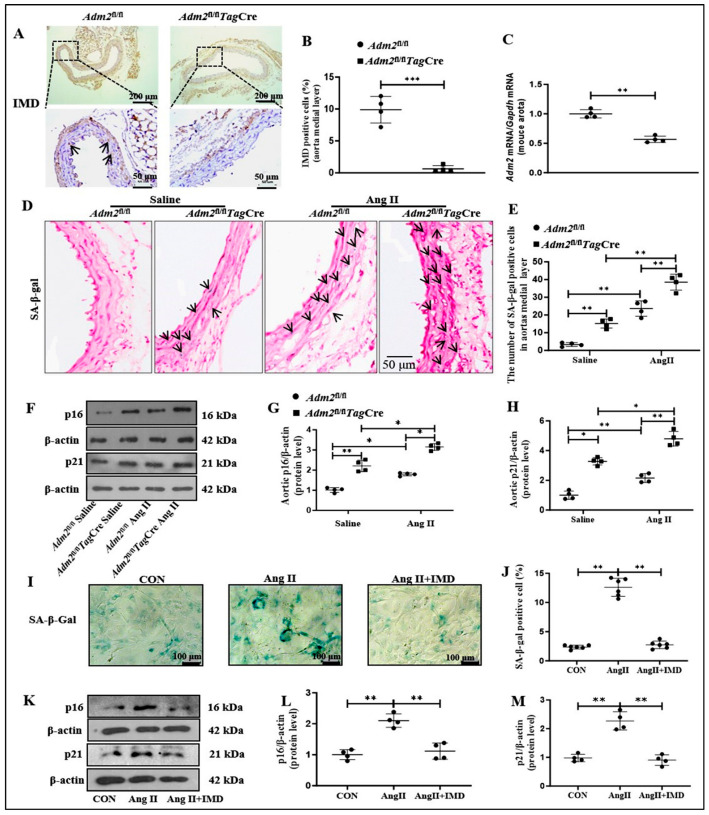
IMD alleviated senescent phenotype transition in tunic media of aorta in mice and in VSMC induced by Ang II. Immunohistochemistry staining for IMD (**A**) the arrows indicated the corresponding positive staining for IMD (brown, scale bar = 200 μm, 50 μm) in the mouse thoracic aortas, and quantification of IMD-positive staining in the medial layer of mouse thoracic aortas ((**B**), *n* = 4). Data are mean ± SD. *** *p* < 0.0001, the Mann–Whitney test. Quantitative RT-PCR analysis of mRNA level of *Adm2* in mouse aortas ((**C**), *n* = 4). Data are mean ± SD. ** *p* < 0.01, the Mann–Whitney test. SA-β-gal staining for β-galactosidase activity (SA-β-gal, (**D**) the arrows indicated the corresponding positive staining for SA-β-gal (blue), scale bar = 50 μm) in the mouse thoracic aortas, and the number of positive cells of β-galactosidase-staining under the same vascular cross-sectional area ((**E**), *n* = 4). Data are mean ± SD. ** *p* < 0.01, the Kruskal–Wallis test. Western blot analysis of protein levels of cyclin-dependent kinase inhibitors p16 and p21 in mouse aortas, and quantification ((**F**–**H**), *n* = 4). Data are mean ± SD. * *p* < 0.05, ** *p* < 0.01, the Kruskal–Wallis test. SA-β-gal staining (blue) (scale bar = 100 μm) and quantification of SA-β-gal-positive staining in mouse VSMCs ((**I**,**J**), *n* = 6). Data are mean ± SD. ** *p* < 0.01, one-way ANOVA. Western blot analysis of protein levels of cyclin-dependent kinase inhibitors p16 and p21 in mouse VSMCs, and quantification ((**K**–**M**), *n* = 4). Data are mean ± SD. ** *p* < 0.01, the Kruskal–Wallis test.

To clarify the role of endogenous IMD in senescent phenotype transition of VSMCs in aorta, we evaluated whether SMCs-specific ablation of IMD would exacerbate Ang II-induced senescent phenotype transition of VSMCs in the aorta of mice. Immunohistochemistry staining verified that IMD was deficient in the aortic media layer of *Adm2*^fl/fl^*Tag*Cre mice (Figure 2A,B). Moreover, the mRNA level of *Adm2* in the aorta of *Adm2*^fl/fl^*Tag*Cre mice was significantly lower than that in the *Adm2*^fl/fl^ group (Figure 2C). Compared with *Adm2*^fl/fl^ Saline mouse aortas, SA-β-gal activity was markedly upregulated in the aortic media layer of *Adm2*^fl/fl^ mice treated with Ang II and further increased in *Adm2*^fl/fl^*Tag*Cre mice treated with Ang II (Figure 2D,E). In addition, Ang II treatment strikingly elevated the protein levels of p16 and p21 in the aorta of *Adm2*^fl/fl^*Tag*Cre mice (Figure 2F–H). These results suggested that IMD deficiency may exacerbate Ang II-induced senescent phenotype transition of VSMCs in aorta. More importantly, SA-β-gal activity and the protein levels of p16 and p21 were also increased in the aorta of the *Adm2*fl/fl*Tag*Cre mice compared to the *Adm2*fl/fl mice. These results suggest that IMD deficiency in VSMCs may exacerbate senescent phenotype transition and further promoted Ang II-induced senescent phenotype transition of VSMCs in aorta.

Next, we applied exogenous IMD_1-53_ to observe the effect of IMD on the senescent phenotype transition of VSMCs in vitro. SA-β-gal staining showed that Ang II treatment significantly increased SA-β-gal activity in VSMCs isolated from *Adm2*^fl/fl^ mice, while exogenous IMD_1-53_ significantly decreased SA-β-gal activity induced by Ang II (Figure 2I,J). In addition, the protein levels of p16 and p21 were significantly increased in Ang II-treated VSMCs compared to the control VSMCs, which was reversed by IMD_1-53_ supplementation (Figure 2K–M). Taken together, these results suggested that IMD could also inhibit Ang II-induced senescent phenotype transition of VSMCs in vitro.

### 2.3. Deficiency in Endogenous IMD Exacerbated Ang II-Induced Vascular Fibrosis and Prosenescent Factors Expression

**Figure 3:**
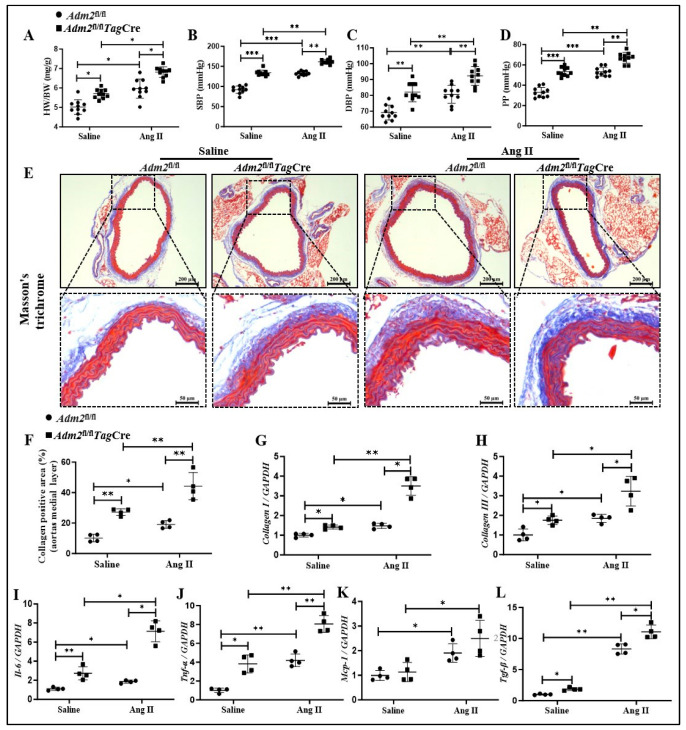
The effects of IMD deficiency on heart weight/body weight (HW/BW), hemodynamic data, aging-related vascular fibrosis, and the levels of senescence-associated secretory Phenotype. No significant changes in heart-to-weight ratio (**A**) and hemodynamic data in IMDSMC^−/−^ mice (**B**–**D**). Systolic blood pressure (SBP), diastolic blood pressure (DBP), and pulse pressure (PP) of mice (*n* = 10) Data are mean ± SD, * *p* < 0.05, ** *p* < 0.01 and *** *p* < 0.001, two-way ANOVA. Masson’s trichrome staining for collagen (blue) in the medial layer of mouse thoracic aortas (scale bar = 200 um, 50 um), and quantification of collagen-positive staining ((**E**,**F**), *n* = 4). Quantitative RT-PCR analysis of mRNA levels of *Col1a1* and *Col3a1* in mouse aortas ((**G**,**H**), *n* = 4). Quantitative RT-PCR analysis of mRNA levels of interleukin-6 (*Il-6*), tumor necrosis factor-α (*Tnf-α*), monocyte chemotactic protein-1 (*MCP-1*), and transforming growth factor-β (*Tgf-β*) in mouse aortas ((**I**–**L**), *n* = 4). Data are mean ± SD, * *p* < 0.05 and ** *p* < 0.01, the Kruskal–Wallis test.

Given that collagen synthesis is upregulated in the progression of vascular aging, which contributes to vascular fibrosis and arterial stiffening [2], we further explored the effects of IMD on blood pressure and vascular fibrosis in the mouse vascular aging model. The results showed that heart weight-to-body weight ratio (HW/BW), systolic blood pressure (SBP), diastolic blood pressure (DBP), and pulse pressure (PP) were significantly increased in *Adm*2fl/fl*Tag*Cre mice and in the Ang II-treated groups (Figure 3A–D), and their levels were more significant in Ang II-treated *Adm*2fl/fl*Tag*Cre mice. Compared to the *Adm2fl/fl* mice, collagen deposition determined by Masson’s trichrome staining was significantly increased in the aortic media layer in *Adm*2fl/fl*Tag*Cre mice and was further elevated in *Adm2*fl/fl*Tag*Cre mice treated with Ang II (Figure 2E,F). Consistent with the above results tendency, the mRNA levels of *Col1a1* and *Col3a1* were also notably increased in the *Adm*2fl/fl*Tag*Cre mice compared with the *Adm2*fl/fl mice, and their levels induced by Ang II in the aortas of *Adm*2fl/fl*Tag*Cre mice were significantly increased compared with those of *Adm2*fl/fl mice (Figure 3G,H).

In addition, prosenescent factors such as interleukin-6 (IL-6), tumor necrosis factor-α (TNF-α), monocyte chemotactic protein-1 (MCP-1), and transforming growth factor-β (TGF-β) are upregulated in aging blood vessels and exert a crucial effect on the pathogenesis of vascular aging [6]. In the present study, real-time PCR results showed that the mRNA levels of *Il-6*, *Tnf-α* and *Tgfb1* were markedly increased in the *Adm2*^fl/fl^*Tag*Cre mice compared with the *Adm2*^fl/fl^ mice, Ang II infusion further increased their levels (*Il-6*, *Tnf-α*, *Mcp-1* and *Tgfb1*) in the aortas of *Adm2*^fl/fl^*Tag*Cre mice (Figure 3I–L). The above results showed that IMD deficiency in VSMCs or Ang II treatment significantly increased blood pressure and aggravated vascular fibrosis and prosenescent factor expression in arterial media, and IMD deficiency in VSMCs significantly promoted Ang II-induced changes, suggesting that IMD deficiency may aggravate vascular senescent phenotype transition and promote the elevation in blood pressure and myocardial hypertrophy.

### 2.4. IMD Alleviated DNA Damage in Ang II-Treated Aortas

**Figure 4:**
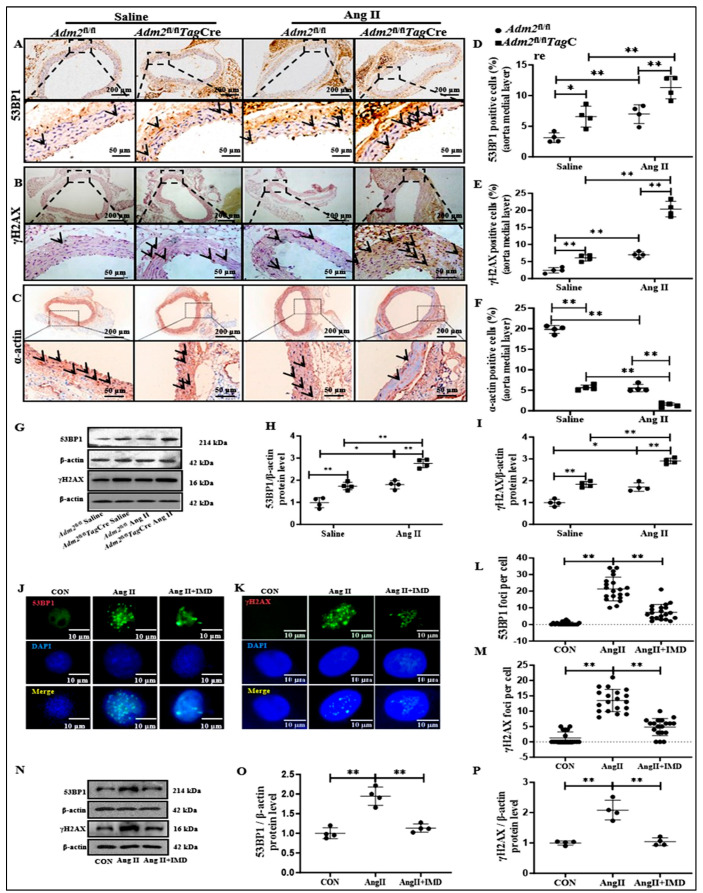
SMC-specific ablation of IMD aggravated DNA damage in aortic senescent phenotype transition induced by Ang II. Immunohistochemistry staining for p53-binding protein 1 (53BP1) ((**A**,**B**), scale bar = 200 μm, 50 μm), p-H2A histone family member X (S139) (γH2AX) and VSMCs phenotype protein α-actin ((**A**–**C**), the arrows indicated the corresponding positive stainings for 53BP1, (A); γH2AX, (B); and α-actin, (C)), scale bar = 200 μm, 50 μm), and quantification of 53BP1-positive staining, γH2AX-positive staining and α-actin-positive staining ((**D**–**F**), *n* = 4) in the medial layer of mice thoracic aortas. Data are mean ± SD. * *p* < 0.05, ** *p* < 0.01, the Kruskal–Wallis test. Western blot analysis of the protein levels of poly (ADP-ribose) polymerase-1 (PARP1), 53BP1, and γH2AX in mouse aortas, and quantification ((**G**–**I**), *n* = 4). Data are mean ± SD. * *p* < 0.05, ** *p* < 0.01, the Kruskal–Wallis test. IMD_1-53_ inhibited Ang II-induced VSMC senescent phenotype transition and DNA damage in vitro. Immunofluorescence staining for 53BP1 and γH2AX foci (green) in mouse VSMCs ((**J**,**K**)). Nuclei were stained with Hoechst 33342 (blue). Merged images are shown (scale bar = 10 μm). Quantitation of the number of 53BP1 and γH2AX foci formation per cell ((**L**,**M**), *n* = 20, one-way ANOVA). Data are mean ± SD. ** *p* < 0.01, the Kruskal–Wallis test. Western blot analysis of 53BP1 and γH2AX protein levels in mouse VSMCs, and quantification ((**N**–**P**), *n* = 4). Data are mean ± SD. ** *p* < 0.01, the Kruskal–Wallis test.

DNA damage is one of the most important causes of vascular aging [6]. The inhibitory effect of IMD on Ang II-induced vascular senescent phenotype transition may be achieved by alleviating DNA damage. We explored the effects of IMD on DNA damage in the Ang II-induced mouse vascular senescent phenotype transition model. Both p-H2A histone family member X (S139) (γH2AX) and p53-binding protein 1 (53BP1) are widely used biomarkers of DNA damage [20,21]. Immunohistochemistry results showed that Ang II treatment significantly elevated the protein levels of 53BP1 and γH2AX in *Adm2*^fl/fl^ mice, and this effect was further strengthened in *Adm2*^fl/fl^*Tag*Cre mice (Figure 4A,B,D,E). Moreover, the increased protein levels of 53BP1 and γH2AX in the arteries of *Adm2*^fl/fl^*Tag*Cre mice were confirmed by Western blot (Figure 4G–I), suggesting that IMD deficiency aggravated DNA damage in mouse aortas. In addition, the protein expression of α-actin of VSMCs phenotype was decreased in *Adm2*^fl/fl^ mice, and this decrease was more significant in *Adm2*^fl/fl^*Tag*Cre mice after Ang II treatment (Figure 4C,F). This further indicated that the VSMCs in the arterial media underwent a phenotypic transformation.

The effect of IMD on DNA damage was further confirmed in Ang II-induced senescent VSMCs in vitro. Consistent with the results in vivo, protein levels of 53BP1 and γH2AX in the nucleus of senescent VSMCs were significantly higher than those in the control group and were decreased under IMD_1-53_ treatment (Figure 4H–K). Similarly, exogenous IMD_1-53_ decreased the protein levels of 53BP1 and γH2AX in Ang II-treated VSMCs (Figure 4L–N). The above results suggested that IMD_1-53_ effectively alleviated DNA damage in senescent VSMCs.

### 2.5. IMD Inhibited DNA Damage and the Senescent Phenotype Transition of VSMCs by Activating PARP1

**Figure 5:**
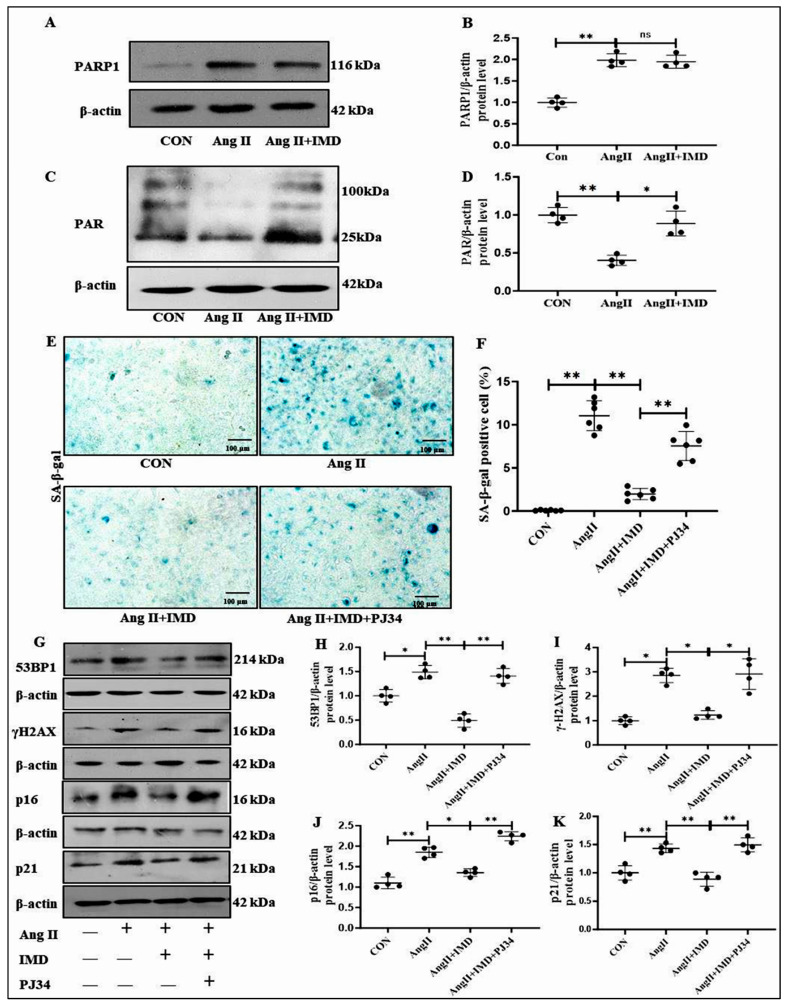
Pharmaceutical inhibition of PARP1 blocked the inhibitory effects of IMD on DNA damage in VSMCs treated by Ang II. Western blot analysis of PARP1 protein level in mouse VSMCs, and quantification ((**A**,**B**), *n* = 4). Data are mean ± SD. ns, no significance. The Kruskal–Wallis test. Western blot analysis of the protein level of poly (ADP-ribose) polymer (PAR) in mouse VSMCs and quantification ((**C**,**D**), *n* = 4). * *p* < 0.05, ** *p* < 0.01, the Kruskal–Wallis test. SA-β-gal staining for β-galactosidase activity (blue) (scale bar = 100 μm) in mouse VSMCs, and quantification of SA-β-gal-positive staining in mouse VSMCs ((**E**,**F**), *n* = 6). Data are mean ± SD. ** *p* < 0.01, one-way ANOVA (**F**). Western blot analysis of the protein levels of p16, p21, 53BP1 and γH2AX in mouse VSMCs, and quantification ((**G**–**K**), *n* = 4). Data are mean ± SD. * *p* < 0.05, ** *p* < 0.01, the Kruskal–Wallis test.

Previous studies have found that IMD upregulates the activity of NAD^+^-dependent deacetylases and plays a protective role in CVDs [15], whereas PARP1 is also an NAD^+^-dependent ADP-ribose polymerase and plays an important role in recognizing DNA damage and initiating DNA damage repair [22]. Therefore, we further investigated whether IMD could inhibit DNA damage and thus reduce vascular senescent phenotype transition by regulating PARP1. Western blot results showed no significant effect of IMD on the protein level of PARP1 in senescent VSMC induced by Ang II (Figure 5A,B). However, compared with the control group, poly ADP-ribose (PAR) protein level in Ang II-treated VSMCs was significantly decreased, while IMD_1-53_ restored PAR level induced by Ang II (Figure 5C,D), suggesting that IMD_1-53_ can significantly increase PARP1 activity. PJ34, an inhibitor of PARP1, was used to explore whether IMD repaired DNA damage by activating PARP1. In Ang II-treated VSMCs, IMD_1-53_ administration significantly inhibited SA-β-gal activity (Figure 5E,F) and decreased the protein levels of p16 and p21, while PJ34 treatment blocked the above effects of IMD_1-53_ (Figure 5G,J,K). Consistently, IMD_1-53_ treatment significantly decreased the protein levels of γH2AX and 53BP1 in Ang II-treated VSMCs, which were blocked by PJ34 (Figure 5G–I). Therefore, these results suggested that IMD_1-53_ could inhibit DNA damage and reverse VSMC senescent phenotype transition by increasing the activity of PARP1.

### 2.6. IMD Increased PARP1 Activity by Activating NAMPT

**Figure 6:**
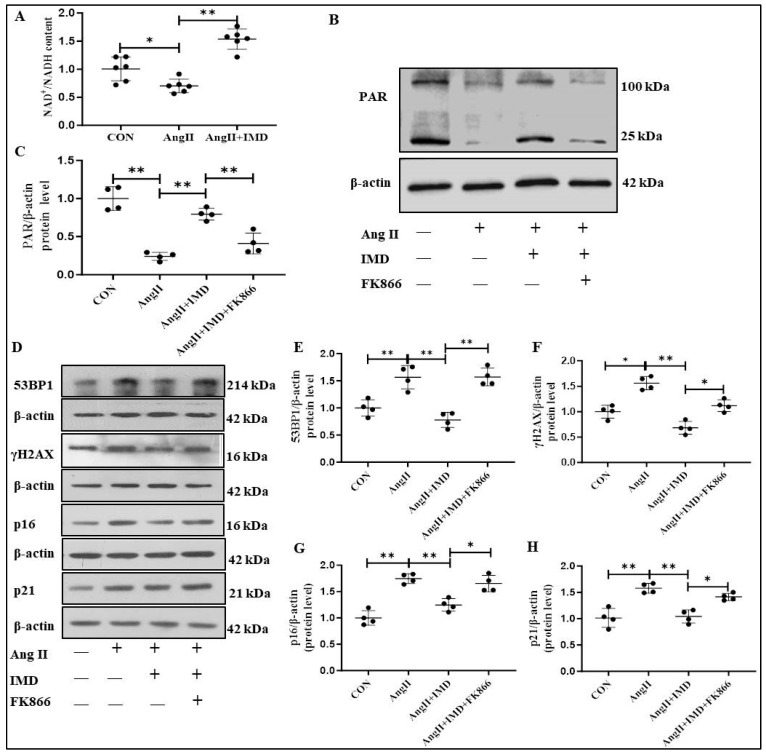
Inhibition of NAMPT blocked the effect of IMD on increasing PARP1 activity in VSMCs treated by Ang II. Quantification of NAD^+^/NADH in mouse VSMCs induced by Ang II and IMD_1-53_ ((**A**), *n* = 4). Data are mean ± SD. * *p* < 0.05, ** *p* < 0.01, one-way ANOVA. Western blot analysis of the protein level of PAR in mouse VSMCs and quantification ((**B**,**C**), *n* = 4). Data are mean ± SD. ** *p* < 0.01, the Kruskal–Wallis test. Western blot analysis of 53BP1, γH2AX, p16 and p21 protein levels in mouse VSMCs, and quantification ((**D**–**H**), *n* = 4). Data are mean ± SD. * *p* < 0.05 and ** *p* < 0.01, the Kruskal–Wallis test.

PARP1 activity relies on nicotinamide adenine dinucleotide (NAD^+^), and the nicotinamide phosphoribosyl transferase (NAMPT)-mediated salvage pathway is a major source of NAD^+^ in VSMCs [23]. In Ang II-treated VSMCs, the ratio of NAD^+^/NADH was significantly reduced, which was reversed by IMD_1-53_ (Figure 6A). Next, the NAMPT inhibitor FK866 was used to explore whether the effect of IMD_1-53_ on NAD^+^ increase was dependent on NAMPT. The protein level of PAR represented the activity of PARP1, which was significantly decreased in the Ang II treatment group. IMD_1-53_ increased the PAR protein level in Ang II-treated VSMC, which was blocked by FK866 (Figure 6B,C), suggesting that NAMPT was involved in IMD_1-53_ mediated PAR production. In addition, IMD_1-53_ treatment significantly decreased the protein levels of γH2AX and 53BP1, which were blocked by FK866 (Figure 6D–F). In addition to DNA damage markers, Ang II-induced elevated protein levels of p16 and p21 were strikingly decreased by IMD_1-53_, but this effect was also blocked by FK866 (Figure 6D,G,H), suggesting that NAMPT was involved in the role of IMD in improving vascular senescent phenotype transition. Collectively, these results suggested that IMD may increase PARP1 activity by activating NAMPT.

## 3. Discussion

Despite intensive studies on the cardiovascular protective function of IMD, its role in the pathogenesis of senescent phenotype transition of VSMCs has not been investigated. In this study, we provided novel evidence that IMD reversed the senescent phenotype transition of VSMCs in tunica media of aorta. Ang II-treated mice showed vascular senescent phenotype transition and decreased IMD expression in tunica media. SMC-specific IMD-deficiency exacerbated senescent phenotype transition of VSMCs and further promoted Ang II-induced transition of VSMCs, while exogenous IMD_1-53_ significantly ameliorated Ang II-induced senescent phenotype transition of VSMCs. Mechanistically, IMD enhanced the activity of PARP1 by activating NAMPT in VSMCs and increasing the levels of NAD^+^ in VSMCs, alleviating DNA damage and then delaying VSMC senescent phenotype transition.

Stress-induced premature senescence is related to CVDs. Ang II, the most potent component of the renin–angiotensin system, plays a central role in cardiovascular aging. A previous study showed that the Ang II concentration increased with age in nonhuman primates [24]. Moreover, Ang II has been shown to induce premature senescence in VSMCs and aortas [17,25]. Therefore, we used Ang II to construct a mouse model with the senescent phenotype transition of VSMCs in tunica media of aorta in mice. Our results showed that Ang II treatment increased the protein levels of p16 and p21 and the activity of SA-β-gal in the aortas of mice, indicating that VSMCs transition from contractile phenotype to senescent phenotype was successfully constructed. Additionally, increased collagen deposition and prosenescent factor levels further confirmed the vascular senescent phenotype transition induced by Ang II. We also used Ang II to induce VSMCs senescent phenotype transition in vitro, and the SA-β-gal activity and the protein levels of p16 and p21 were significantly increased in Ang II-treated VSMCs compared to the control group, suggesting that Ang II successfully induced senescent phenotype transition of VSMCs. Previous studies showing that high concentrations of Ang II may lead to VSMC apoptosis were considered [26].

IMD is a cardiovascular polypeptide involving the maintenance of cardiovascular homeostasis, and it exerts a potential beneficial role in CVDs. Increasing evidence shows that IMD has a protective effect on vascular abnormalities, including vascular calcification, vascular inflammation, and abdominal aortic aneurysm which all involve the process of vascular senescence [14,15,16,27,28]. A previous study showed that IMD was downregulated in naturally aged rat aortas [15]. In this study, IMD mRNA level was significantly decreased in the aortas of mice with vascular aging induced by Ang II. In the *Adm2*^fl/fl^*Tag*Cre mice, we found that IMD deficiency in VSMCs promoted prosenescent factor expression (*Il-6*, *Tnf-α* and *Tgfb1*), inhibited the expression of VSMCs marker protein (α-actin), and aggravated vascular senescent phenotype transition, which might indicate that IMD deficiency in VSMCs aggravated the elevation in BP and vascular fibrosis by promoting vascular aging in tunica media of aorta. Moreover, exogenous IMD_1-53_ administration ameliorated VSMCs senescent phenotype transition. These results suggest that IMD in the aortas may play an important role in inhibiting vascular senescent phenotype transition and further alleviating aging.

DNA damage is a critical characteristic and mechanism of cellular senescence [29,30]. Nuclear and mitochondrial DNA damage genomes both have been found in aging mammals [31,32]. 53BP1 and γH2AX are two general markers of DNA damage. When DNA double-strand damage occurs, the S139 site of histone H2AX is phosphorylated to form γH2AX [33]. 53BP1 is an important regulatory molecule of DNA double-strand damage, which can rapidly aggregate to chromatin near the damage site [34,35]. Our data showed the increased protein levels of 53BP1 and γH2AX both in Ang II-treated aortas and VSMCs, which were further enhanced in IMD deficient mice, and were ameliorated by exogenous IMD_1-53_ treatment in vitro. These results suggest that IMD may alleviate vascular senescent phenotype transition in tunica media by alleviating DNA damage.

Poly ADP-ribosylation is an immediate repair response to mitigate DNA damage and is catalyzed primarily by PARP1 [36,37]. After PARP1 recognizes DNA damage, it catalyzes the formation of PAR, which plays an important role in recruiting subsequent effector molecules [22]. Thus, the PAR protein level reflects the PARP1 activity [38,39]. PARP1 mitigated replicative cell senescence by activating homologous recombinant double-strand rupture repair [40]. Therefore, we speculated that PARP1 might be involved in the beneficial effect of IMD on Ang II-induced vascular senescent phenotype transition. We found that the protein level of PARP1 was significantly increased in Ang II-treated aortas and VSMCs, which suggested a critical role of PARP1 in Ang II-induced vascular senescent phenotype transition, indicating that the compensatory increase in PARP1 may be in response to the DNA damage induced by Ang II. Although exogenous IMD_1-53_ did not significantly regulate the protein level of PARP1 in VSMCs, IMD might promote DNA repair by increasing PARP1 activity. As previously reported, Checkpoint kinase 2 (CHK2) increases PARP1 activity, promoting the repair of oxidative DNA damage without influencing PARP1 protein expression [41]. Our data showed that the protein level of PAR was significantly elevated in the IMD_1-53_ treatment group, suggesting that IMD might increase PARP1 activity. Furthermore, the protective effects of IMD_1-53_ against DNA damage and VSMC senescent phenotype transition were blocked by the PARP1 inhibitor PJ34. These results suggested that IMD might inhibit VSMCs senescent phenotype transition by enhancing PARP1 activity.

The activity of PARP1 to catalyze the formation of PAR is NAD^+^-dependent [36]. NAD^+^ is synthesized through two pathways, namely the de novo pathway from amino acids and the salvage pathway from nicotinamide, and the latter is the main source of NAD^+^ in mammalian cells [42]. NAMPT is the rate-limiting enzyme of the salvage pathway and regulates the intracellular NAD^+^ pool, participating in the regulation of NAD^+^-dependent enzyme activity [23]. Reducing the cellular NAD^+^ content with the NAMPT inhibitor FK866 can significantly reduce the activity of PARP1. Consistently, our data showed that the relative content of NAD^+^ in Ang II-treated VSMCs was significantly decreased, which was significantly increased after IMD_1-53_ administration. In addition, the NAMPT inhibitor FK866 successfully blocked the effect of IMD_1-53_ on enhancing PARP1 activity and alleviating DNA damage. These results suggested that IMD might activate PARP1 by enhancing the activity of NAMPT. However, the specific molecular mechanism by which IMD regulates NAMPT warrants further exploration.

Previous studies demonstrated that Ang Ⅱ-induced hypertension and myocardial hypertrophy in mice could be improved by systemic IMD_1-53_ treatment [43]. Our present study indirectly showed that IMD located in the medial layer of the artery might have the ability to exert the anti-hypertensive effect through inhibiting Ang Ⅱ-induced remodeling of the arterial media. Mechanistically, the IMD loss of VSMCs might promote the transformation of the aging phenotype of VSMCs that further contribute to vascular remodeling, thereby increasing blood pressure and myocardial hypertrophy. One limitation of the current study is the absence of functional data in assessing vascular reactivity. For instance, the measurements of acetylcholine-induced vasodilation would strengthen the evaluation of endothelial cell function, which can further support the protective role of IMD in vascular tissue. Another limitation is that only using male animals for exploring the effect of IMD on the vascular senescent phenotype transition is insufficient. Therefore, we will pay attention to these limitations and improve the relevant experiment in future research.

In conclusion, our study discovered a novel mechanism that IMD can improve vascular senescent phenotype transition in tunica media by alleviating DNA damage. Mechanistically, IMD enhanced PARP1 activity by increasing NAMPT-mediated NAD^+^ production, resulting in the promotion of DNA damage repair and improvement in VSMCs senescent phenotype transition. This study uncovered the beneficial effect of an endogenous active peptide on vascular senescent phenotype transition in tunica media, which may provide a new target for the prevention and therapy of vascular aging-related diseases.

## 4. Materials and Methods

### 4.1. Materials

Antibodies for IMD (Sc-86272), β-actin (Sc-47778), and all secondary antibodies (horseradish peroxidase-conjugated anti-mouse, anti-rabbit, or anti-goat IgG) were from Santa Cruz Biotechnology (Santa Cruz, CA, USA). Antibodies for poly [ADP-ribose] polymer (PAR, Ab14459), p21 (Ab109199), p16INK4a (p16, Ab189034), 53BP1 (Ab36823), and p-H2A histone family member X (S139) (γH2AX, Ab26350) were from Abcam (Cambridge, UK). Antibodies for α-actin and PARP1 (CST9532) were from Cell Signaling Technology (Danvers, MA, USA). The nitrocellulose membrane was from Amersham Life Science (Amersham, UK). PJ34 (HY-13688A) was from MedChemExpress (Monmouth Junction, NJ, USA). FK866 (A4381) was from Apexbio (Houston, TX, USA). The sequences of oligonucleotide primers for Real-time PCR amplification were synthesized by Tsingke Biotechnology (Beijing, China). TRIzol reagent and the enhanced chemiluminescence (ECL) kit were from Applygen Technologies (Beijing, China). The SuperReal PreMix Plus (SYBR Green) kit and The FastKing RT Kit (with gDNase) for Real-time PCR were from Tiangen Biotech (Beijing, China). Alzet mini-osmotic pumps were from DURECT Corp (model 1004, Cupertino, CA, USA). Synthetic human IMD_1-53_ and human angiotensin II (Ang II) were from Phoenix Pharmaceuticals (Belmont, MA, USA). Other chemicals and reagents were of analytical grade.

### 4.2. Animals

All animal care and experimental protocols complied with the Guide for the Care and Use of Laboratory Animals published by the US National Institutes of Health (NIH Publication, 8th Edition, 2011) and passed verification by the Animal Care Committee of Peking University Health Science Center. *Adm2*^fl/fl^*Tag*Cre mice were generated on a C57BL/6 background from the Animal Research Center of Nanjing University (Cat. NO.131110, Nanjing, China) and were previously generated by crossing mice carrying loxP-flanked *Adm2* alleles with *Tagln*-Cre transgenic mice (C57BL/6, Cat. NO. NM-KI-200144, Shanghai, China). Mice (male) were randomly assigned to 4 groups (n = 10 each group): *Adm2*^fl/fl^ Saline, *Adm2*^fl/fl^*Tag*Cre Saline, *Adm2*^fl/fl^ Ang II, and *Adm2*^fl/fl^*Tag*Cre Ang II. For the vascular aging model, 12- to 14-week-old male *Adm2*^fl/fl^*Tag*Cre mice and their age-matched *Adm2*^fl/fl^ mice underwent the application of saline or Ang II (dissolved in saline) by subcutaneous implantation of an Alzet osmotic minipump. Saline or Ang II (1.44 mg/kg/d) were then continuously infused for 4 weeks [17]. All animals were fed normal mouse chow. After 4 weeks, blood pressure was measured in conscious mice by the standard tail-cuff methods as described [18]. At the end of the experiment, mice were anesthetized by using 3% isoflurane administration via inhalation through a mask. After anesthetization, all mice were killed by exsanguination, and the aortas were quickly removed for further analysis.

### 4.3. Senescence-Associated β-Galactosidase Staining

Senescence-associated β-galactosidase (SA-β-gal) staining was performed using a commercial kit (#9860S, Cell Signaling Technology, Danvers, MA, USA). Segments of mouse thoracic aortas were freshly placed in optimal cutting temperature compound and cut into 7-μm-thick sections. Aorta sections or VSMCs were fixed by using 4% paraformaldehyde for 15–20 min, washed with PBS, incubated in β-galactosidase staining solution at 37 °C for 12 h, and then Eosin re-staining was conducted and photographed. Staining data were quantified by using Image-Pro Plus v6.0.

### 4.4. Masson’s Trichrome Staining

Segments of mouse aorta (1 cm) were placed in 4% phosphate buffered neutral formalin (pH 7.4, 0.1 mol/L) for 12 h and then immersed in 20% sucrose solution for storage. Aorta samples were dehydrated and embedded in paraffin, cut into 5-μm-thick sections, and then subjected to Masson’s trichrome staining (G6004, Servicebio, Wuhan, China). For Masson’s trichrome staining, the arterial media was delineated and the percentage of media positive for blue staining was assessed by using Image-Pro Plus v6.0 (Media Cybernetics, Rockville, MD, USA).

### 4.5. Immunostaining

Segments of mouse thoracic aortas embedded in paraffin underwent immunohistochemical staining. Sections (5 μm) were incubated with antibodies against IMD (1:40), 53BP1 (1:500), γH2AX (1:100), or α-actin (1:200) at 4 °C overnight, and then with secondary antibody for 1 h at 37 °C. Nuclei were stained with hematoxylin and then treated with diaminobenzidine. Immunostaining data were quantified by using Image-Pro Plus v6.0. VSMCs were first fixed in 4% paraformaldehyde for 15 min and permeabilized with 0.3% Triton X-100 for 10 min. Nonspecific binding was reduced by incubation in 1% bovine serum albumin diluted in PBS for 60 min at 37 °C. VSMCs were incubated with antibodies against 53BP1 (1:200) or γH2AX (1:100) at 4 °C overnight, rinsed with PBS and incubated with fluorescein-labeled secondary antibodies. Nuclei were stained with Hoechst 33342 (Sigma-Aldrich, St. Louis, MO, USA). Images were acquired under fluorescence microscopy (Leica, Cambridge, UK).

### 4.6. Real-Time (RT) PCR Analysis

Total RNA from aortic tissues was isolated and reverse transcribed. RT-PCR amplification was performed using the Applied Biosystems 7500 fast PCR System (Waltham, MA, USA) and SYBR Green I reagent (#FP205-02, Tiangen Biotech, Beijing, China). The cycle threshold (Ct) was determined as the number of PCR cycles required for a given reaction to reach an arbitrary fluorescence value within the linear amplification range. Relative quantification was performed according to the 2^−δΔCt^ method with the GAPDH level as a reference. The forward and reverse PCR primers sequences and annealing temperature were listed as follows in Table 1.

### 4.7. Western Blot Analysis

Extracts of aortic tissues or cells containing equal amounts of total protein were resolved by 10% or 12% SDS-PAGE and then transferred to a nitrocellulose membrane. Nonspecific proteins were blocked with 5% nonfat dried milk for 1 h, and then incubated with primary antibodies for β-actin (1:3000), 53BP1 (1:5000), γH2AX (1:500), p21 (1:500), p16 (1:500), PARP1 (1:1000), and PAR (1:1000) overnight at 4 °C. The secondary antibody (1:2000) was incubated for 1 h at room temperature. The labeling of proteins was shown by enhanced chemiluminescence. The amounts of proteins were analyzed by using NIH ImageJ (v1.8.0) and normalized to β-actin expression.

### 4.8. Cell Culture

Male mouse VSMCs were isolated from 8-week-old C57BL/6J mice as described in [14]. In brief, thoracic aortas were digested by using 1 mg/mL type II collagenase (#LS004176, Worthington Biochemical Corp, Lakewood, NJ, USA) at 37 °C for 10–15 min. The adventitia and endothelium were removed. Aortas were placed in DMEM containing 10% FBS, 100 U/mL penicillin, and 100 μg/mL streptomycin overnight in a 37 °C incubator with 5% CO_2_, and then digested in 5 mL of 1 mg/mL type II collagenase and 0.25 mg/mL elastase I (#E1250, Sigma-Aldrich, St. Louis, MO, USA) at 37 °C for 90 min. After centrifugalization at 1000 rpm for 3 min, the cells were cultured with DMEM containing 20% FBS with 5% CO_2_ at 37 °C. VSMCs at passage 5–7 were used for the relevant experiments. As previously described in [14], confluent VSMCs were incubated in DMEM containing 10% FBS, 100 μg/mL streptomycin, and 100 U/mL penicillin, treated with Ang II (10^−7^ mol/L) or IMD_1-53_ (10^−7^ mol/L), and cultured at 37 °C in an incubator containing 5% CO_2_ and 95% air. After treatment for 3 days, the cells were collected for the relevant experiments. To explore the signaling pathway by which IMD_1-53_ involves VSMC senescence, VSMCs were preincubated with the PARP1 inhibitor PJ34 (5 μmol/L) or the NAMPT inhibitor FK866 (5 μmol/L) for 30 min.

### 4.9. Intracellular NAD^+^/NADH Content

Total intracellular NAD^+^/NADH was assessed using the NAD^+^/NADH detection kit (S0175, Beyotime, Shanghai, China) referring to the manufacturer’s instructions. VSMCs were collected and directly lysed in lysis buffer from the detection kit. Total NAD and NADH in the extract were detected by colorimetry with a microplate reader (iMARK, BioRad, Hercules, CA, USA).

### 4.10. Statistical Analysis

All data are expressed as mean ± SD and were analyzed by GraphPad Prism 9.20 (GraphPad Software Inc., San Diego, CA, USA). The Kolmogorov–Smirnov test was used to evaluate the normality of the data distribution (*p* > 0.1), and the F-test was used to compare variances (*p* > 1 take for equal variance). All data showed normal distribution and passed equal variance testing. When dealing with small sample sizes (*n* = 4), Mann–Whitney test for the analysis of two groups or the Kruskal–Wallis test for comparing more than two independent groups were used. When dealing with sample sizes (*n* = 4), the unpaired Student’s *t*-test was used to identify significant differences between the two groups (*n* > 4). Comparisons of more than two groups in cellular experiment were analyzed by one-way analysis of variance (ANOVA, *n* > 4). Differences among the different animal groups were evaluated using two-way ANOVA (*n* > 4). When significant differences were detected by ANOVA, Tukey’s test was used for multiple comparisons. Statistical significance was accepted at *p* < 0.05.

## Figures and Tables

**Table 1:** The forward and reverse PCR primers sequences and annealing temperature.

Targets	Sequence		Temp (°C)
*Adm2*	Forward	5-CTTGCCAGCTGTCTCCAGAT-3′	60
	Reverse	5′-CAGGTAGAGGAGGCTGATGC-3′	
*Col1a1*	Forward	5′-GCTCCTCTTAGGGGCCACT-3′	60
	Reverse	5′-CCACGTCTCACCATTGGGG-3′	
*Col3a1*	Forward	5′-CTGTAACATGGAAACTGGGGAAA-3′	60
	Reverse	5′-CCATAGCTGAACTGAAAACCACC-3′	
*Tgfb1*	Forward	5′-GGCACCATCCATGACATGAACCG-3′	60
	Reverse	5′-GCCGTACACAGCAGTTCTTCTCTG-3′	
*Il-6*	Forward	5′-AGGAGTGGCTAAGGACCAAGACC-3′	60
	Reverse	5′-TGCCGAGTAGACCTCATAGTGACC-3′	
*Mcp-1*	Forward	5′-CTATGCAGGTCTCTGTCACGCTTC-3′	60
	Reverse	5′-CCAGTGAATGAGTAGCAGCAGGTG-3′	
*Tnf-α*	Forward	5′-GCATGATCCGAGATGTGGAACTGG-3′	60
	Reverse	5′-CGCCACGAGCAGGAATGAGAAG-3′	
*Gapdh*	Forward	5′-ACTTTGTCAAGCTCATTTCC-3′	60
	Reverse	5′-TGCAGCGAACTTTATTGATG-3′	

## Data Availability

Data is contained within the article.
